# Emaciated mannequins: a study of mannequin body size in high street fashion stores

**DOI:** 10.1186/s40337-017-0142-6

**Published:** 2017-05-02

**Authors:** Eric Robinson, Paul Aveyard

**Affiliations:** 1 Institute of Psychology, Health & Society, University of Liverpool, Liverpool, L69 7ZA UK; 2 Nuffield Department of Primary Care Health Sciences, University of Oxford, Oxford, OX2 6GG UK

**Keywords:** Mannequins, Thin ideal, Body image, High street fashion

## Abstract

**Background:**

There is concern that the body size of fashion store mannequins are too thin and promote unrealistic body ideals. To date there has been no systematic examination of the size of high street fashion store mannequins.

**Methods:**

We surveyed national fashion retailers located on the high street of two English cities. The body size of ‘male’ and ‘female’ mannequins was assessed by two blinded research assistants using visual rating scales.

**Results:**

The average female mannequin body size was representative of a very underweight woman and 100% of female mannequins represented an underweight body size. The average male mannequin body size was significantly larger than the average female mannequin body size. Only 8% of male mannequins represented an underweight body size.

**Conclusions:**

The body size of mannequins used to advertise female fashion is unrealistic and would be considered medically unhealthy in humans.

## Plain english summary

There is concern that the body size of fashion store mannequins are too thin and promote unrealistic body ideals. Although some retailers have reported that they now use more appropriate sized mannequins, the actual size of mannequins being used in high street fashion stores has not been formally examined. We surveyed national fashion retailers located on the high street of two English cities. The average female mannequin body size was representative of a very underweight woman and all mannequins rated represented an underweight body size. The average male mannequin body size was significantly larger than the average female mannequin body size. The body size of mannequins used to advertise female fashion is unrealistic and would be considered medically unhealthy in humans.

## Background

It is well recognised that internalisation of ‘ultra-thin’ body ideals in women acts as risk factor the development of eating disorders and impaired psychological well-being [1, 2]. However, in the modern developed world unrealistic body ideals are communicated both implicitly and explicitly to women [3]. For example, female models tend to have very thin body sizes that would be unattainable for most women [4, 5]. The body size dimensions of the popular young girls toy doll ‘Barbie’ are also implausibly slim [6]. Similarly, catwalk fashion models often appear to be severely underweight [7] and public concern has resulted in recent legislation in European countries banning the use of very underweight catwalk models [8]. Likewise, the use of underweight models has recently been banned in Israel because of concerns that the use of such models communicates ultra-thin body ideals to young people. Thus, there is growing awareness that prevention efforts against body image problems need to address the wider environment and reduce communication of ultra-thin body ideals [9, 10].

Rintala and Mustajoki [11] assessed six female mannequins made in Italy, Japan and Malaysia between the 1920s and 1960s. Based on the body dimensions of these mannequins, the authors concluded that if a human female had the same body dimensions, she would have such little body fat that she would be unable to menstruate. Of late, there has been public concern that the typical size of high street (the primary retail business street in a city or town) fashion store mannequins used in England represents an unrealistic body size for women [12, 13] and this may communicate inappropriate body size ideals. In response to this there have been some news reports that nationwide fashion retailers in England have started to use larger more realistic mannequin sizes [14]. However, there has been no systematic examination of the size of high street fashion store mannequins. The body size of mannequins used to sell fashion may be relevant to the prevention of body image problems because slender mannequins may constitute an environmental factor that communicates and reinforces the ultra-thin ideal. The aim of the present research was to examine the size of male and female mannequins used in national high street fashion retailers in England. A secondary aim was to examine whether the size of mannequins used in stores differed dependent on the age of consumer a store targeted. We reasoned that stores targeting younger age ranges may be more likely to use slender mannequins because of the greater value attached to thinness in young people [15].

## Methods

### Survey sites

We surveyed national fashion retailers located on the high street of two large cities in the north (Liverpool) and centre (Coventry) of England. ‘High street’ stores were operationalised as stores located on the street that was home to the main shopping promenade area of that city. Stores were eligible to be sampled if they had at least one mannequin on display (male and/or female) and were part of a national chain (i.e. more than one store in the country). We opted to sample only national chains as we reasoned that this sampling approach would produce the most representative data for the size of mannequins widely used in high street fashion. The study procedures used were approved by the first author’s University Research Ethics Committee.

### Measurement of mannequin body size

It was our original intention to collect anthropometric measurements of mannequins. However, we contacted all eligible retailers across both survey sites and no retailers gave permission to take anthropometric measurements. We therefore used visual rating scales to assess mannequin body size.

### Rating scales

**Fig. 1:**
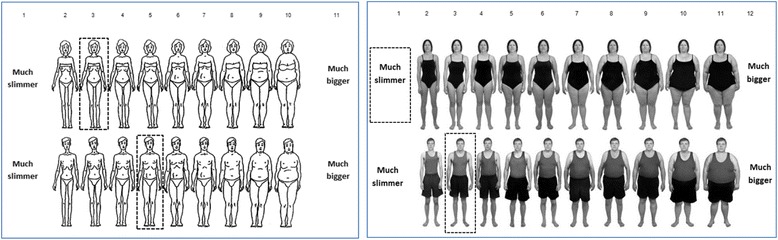
Average size of male and female mannequins. Mean mannequin sizes rated using the Contour Drawing Rating Scale (left) and the BMI-based body size guide rating scale (right) are denoted by broken line boxes. For the BMI-based body size guide rating scale: scale points 1 and 2 (underweight), 3 and 4 (healthy weight), 5 (overweight), 6–12 (class I obesity and above)

When rating mannequins each researcher completed two rating scales by selecting the figure on each scale that most closely resembled the size of mannequin being rated; a BMI-based body size guide rating scale [16] and the Contour Drawing Rating scale [17]. The BMI-based body size guide rating scale consists of ten standardised photographs of adults with known BMI values and is a validated body size perception tool [16]. The scale is gender specific and the photographs range in size from underweight (BMI < 18.5) to class III obesity (BMI ≥ 40), with approximately a 3-point BMI difference between scale figures. For the purpose of this study, we included scale ends marked as ‘much slimmer’ or ‘much bigger’ than the first and last figures in the series, as during piloting we noticed that a number of mannequins were smaller than the first scale figure. This produced a score between 1 and 12. See Fig. 1. The Contour Drawing Rating Scale is a widely used tool to determine visual perceptions of body size [17]. The scale consists of nine male/male front view contour drawings which increase in body size, ranging from a figure which appears very emaciated to a clearly overweight figure. We again included scale ends marked as ‘much slimmer’ and ‘much bigger’ resulting in a rating score between 1 and 11. See Fig. 1. The order in which the two scales were used to make ratings was counterbalanced.

### Rating procedure

Two researcher assistants (1 male, 1 female) blinded to the authors’ hypotheses rated mannequins in both survey sites during September 2015. To be eligible for rating, a mannequin had to have a sufficient amount of its body shape visible, as agreed by the two research assistants (e.g. mannequins wearing long coats, baggy or loose fitting garments were ineligible, whereas mannequins wearing t-shirt and shorts were deemed eligible). On entering a store, the two researchers identified two male and two female mannequins that they both agreed met the above eligibility criteria. Where there were more than two male and two female mannequins eligible, the mannequins that both research assistants agreed were nearest to the store entrance (judged visually) were selected for rating. We opted for this approach because we reasoned that mannequins close to the store entrance would be most visible to shoppers and rating them would minimize the amount of time that researchers spent in stores. After identifying an eligible mannequin, each researcher circled the mannequin, inspected its body size and then rated the mannequin out of view of the other researcher, in order to ensure blinding to each other’s ratings. The order in which male vs. female mannequins were rated was counterbalanced on a store by store basis.

### Analysis strategy

In order to examine the average mannequin size in each store, we averaged each researcher’s rating scores for the two female mannequins and the two male mannequins, before collapsing this rating data across the two researchers. This resulted in each store being allocated an average score for the size of their male mannequins and an average score for their female mannequins. If a store had only one eligible mannequin on display to be rated, we used the rating scores for that mannequin as the store average. We did this separately for ratings made of male and female mannequins so that we could examine whether there were gender differences in mannequin size by conducting t-tests on the male vs. female mannequin rating data.

We also examined whether stores with a younger target age range were more or less likely to use slender mannequins. To do this we asked a sample of 30 members of the general public to classify all of the surveyed shops into one of the following targe﻿t age ranges; under 30 years, 30–50 years, 60 years and above, no specific age range. We classified stores as having a younger target age range (<30 years) if the majority of respondents selected ‘under 30 years’ for that store. We used t-tests to compare mannequin sizes in stores targeting a younger age range vs. all other stores. We also asked the same sample to classify the stores according to their perceived price range, but we did not find that stores were reliably categorised according to perceived price range and therefore did not examine whether mannequin size differed dependent on a store’s perceived price range.

We calculated the percentage of mannequins that would qualify as being ‘underweight’ by whether a mannequin was rated by both research assistants as having an underweight body size, according to the scale points on the BMI-based body size guide rating scale (scale point 1 or 2). We compared percentage of underweight mannequins in female vs. male mannequins using chi-square.

### Inter-rater agreement

Given that the incremental increases in body size across both scales are subtle (see Fig. 1), we examined the percentage of the time that that the two researcher assistants made very similar ratings (i.e. both chose the same scale option or within one scale rating). This figure was 93% using the BMI-based body size guide rating scale and 75% for the Contour Drawing Rating Scale. The overall inter-rater agreement was therefore 84%, which we deemed acceptable because inter-rater agreement scores of ≥ 80% are generally seen to be acceptable in research of relevance to public health [18].

## Results

In total, 17 stores were eligible to be surveyed across the two sites. Sixteen stores displayed at least one eligible female mannequin that was suitable for rating and 15 stores displayed at least one eligible male mannequin suitable for rating. Within those stores 32 female mannequins and 26 male mannequins were rated.

The mean rating for female mannequins on the contour scale was 2.9, between the first and second figure. On the BMI photo scale it was 1.4, lower than the slimmest BMI photo, which has a BMI of < 18.5 kg/m^2^. For male mannequins, the mean rating was 4.8 on the contour scale, between the third and fourth figures, while on the BMI photo scale it was 2.8, between the first and second figures (Table 1), a BMI of approximately 20–22 kg/m^2^.

**Table 1:** Summary data for mannequin size ratings

	Contour drawing rating scale		BMI-based body size guide rating scale	
	Female mannequins	Male mannequins	Female mannequins	Male mannequins
All eligible stores	M = 2.92 CIs = 2.64, 3.21 SD = 0.54 *N* = 16	M = 4.80 CIs = 4.42, 5.18 SD = 0.69 *N* = 15	M = 1.42 CIs = 1.31, 1.54 SD = 0.21 *N* = 16	M = 2.75 CIs = 2.48, 3.02 SD = 0.49 *N* = 15
Stores with < 30 years target market	M = 2.81 CIs = 2.52, 3.09 SD = 0.37 *N* = 9	M = 4.39 CIs = 3.83, 4.96 SD = 0.61 *N* = 7	M = 1.44 CIs = 1.26, 1.63 SD = 0.24 *N* = 9	M = 2.43 CIs = 2.03, 2.82 SD = 0.43 *N* = 7
Stores without < 30 years target market	M = 3.07 CIs = 2.42, 3.72 SD = 0.70 *N* = 7	M = 5.16 CIs = 4.68, 5.62 SD = 0.57 *N* = 8	M = 1.39 CIs = 1.21, 1.57 SD = 0.20 *N* = 7	M = 3.03 CIs = 2.73, 3.34 SD = 0.36 *N* = 8

*M* mean, *CIs* 95% confidence intervals, *SD* standard deviation, *N* number of stores with eligible mannequins

Paired samples t-tests indicated that female mannequins were significantly slimmer than male mannequins using both the contour scale (t (13) = 8.33, *p* < 0.001, mean difference = 1.91) and the BMI photo scale (t (13) = 8.02, *p* < 0.001, mean difference = 1.34).

Independent samples t-tests indicated that for both the contour scale (t (14) = 0.98, *p* = 0.34, d = 0.46) and the BMI scale (t (14) = 0.46, *p* = 0.66, d = 0.23) the size of female mannequins in fashion stores targeting a younger age range (<30 years) vs. all other fashion stores were similar (Table 1). For both the contour scale (t (13) = 2.51, *p* = 0.026, *d* = 1.30) and the BMI scale (t (13) = 2.96, *p* = 0.011, d = 1.51) the size of male mannequins in fashion stores targeting a younger age range was significantly smaller than in stores targeting all other age ranges (Table 1). When examined in mixed 2x2 ANOVAs, the statistical interaction between gender of mannequin and store target age range was significant for the BMI scale (*p* = 0.036), but not for the contour scale (*p* = 0.26).  In addition, because analyses examining the effect of store target age range are reliant on small sample sizes, caution is needed when interpreting findings relating to store target age range. 

Among female mannequins, 100% (32/32) were underweight. Among male mannequins this percentage was 8% (2/26). This difference was statistically significant (*x* ^2^ = 50.4, *p* < .001).

## Discussion

In the present research we examined the size of mannequins used in national fashion retailers on the high streets of two English cities. The average female mannequin body size was representative of a very underweight woman and all female mannequins rated represented an underweight body size for a female human. This was not the case for male mannequins. The average male mannequin body size was significantly larger than the average female mannequin body size and was representative of a healthy weight man. Only 8% of male mannequins represented an underweight body size for a male. We found that female mannequins used by high street fashion stores that target for a younger market (<30 years old) were similarly slender in size, when compared to mannequins used in stores without that target market. However, male mannequins used by high street fashion stores that target a younger age range were significantly slimmer than their equivalents in stores not targeting a younger age range, although analyses regarding store target age range were limited in sample sizes and should be interpreted cautiously.

There have been some news reports of national fashion retailers starting to use more appropriate sized female mannequins in their stores [14] and although we sampled some of these stores in the present study, we found no evidence of appropriate sized female mannequins being used. A potential explanation of the difference in slenderness between female and male mannequins is that the choice of mannequin body sizes by retailers reinforces the idealisation of female slenderness in western culture. Men tend to have a larger ideal body size/weight than women [19, 20]. However, most females would not be likely to desire a body size which would be comparable to the extreme slenderness of mannequins we observed, nor would it be healthy. In addition, the tendency for male mannequins in shops targeting young age ranges to be slimmer in size than other shops may reflect generational shifts in male body size ideals, with slimmer now being more socially acceptable in young men [15].

In the present study we did not formally assess how muscular male mannequins were. Although male mannequins were less likely to be slender than female mannequins and therefore more representative of what constitutes a ‘normal’ male body weight, during data collection it was noted that a number of the male mannequins appeared unrealistically muscular. Men are sometimes presented in the media as muscular and this may have become more extreme over time [21, 22]. In the same way that exposure to ultra-thin ideals may negatively affect body image in women, exposure to unattainable muscular ideals may promote body dissatisfaction in men [23]. Thus, formal examination of whether male mannequins promote unrealistic muscular body ideals for men would now be informative.

A common explanation for why slender female mannequins are used in the fashion industry is because clothes may be more aesthetically pleasing on slender mannequins. However, it is also plausible that using inappropriate sized mannequins may actually be counterproductive to fashion retailers, as consumers may feel that the clothes would not suit their body size [24]. More importantly, it has been shown that internalization of ultra-thin ideals is likely to be detrimental to the psychological well-being of young women [1, 25]. Therefore, environmental factors that highlight or implicitly support the importance of ultra-thinness may be damaging. In two experimental studies, young girls that played with a doll denoting a very slim body size, as opposed to a more appropriate size, reported greater internalisation of thin ideals [26, 27]. Likewise, numerous studies have shown that exposure to media that portrays female thinness can promote body dissatisfaction in women [28] and body dissatisfaction is a risk factor for the development of disordered eating and depressive symptoms [29, 30]. It is important to note that the present research does not tell us whether ultra-thin fashion mannequins have any observable direct effect on body image, but we presume that the widespread use of inappropriate mannequin body sizes may reinforce unrealistic body ideals in some people.

### Limitations

A limitation of the present study was that because stores did not provide us with permission to take objective anthropometric measurements of mannequins, we instead had to rely on subjective visual ratings. Two rating scales were used by two independent raters and results were consistent across both rating scale. Inter-rater agreement was slightly lower than is normally deemed acceptable (74% rather than ≥80%) for one of the two rating scales used, although there was acceptable overall inter-rater agreement. However, the use of objective measurement would be preferential and would allow for more precise measurement. Our sampling procedure involved researchers identifying and rating two mannequins (of each sex) that were closest to the entrance in each eligible store and had a sufficient amount of their ‘body’ visible. We opted for this approach to minimise the amount of time that researchers spent in each store and because during piloting we noted no variability in mannequin sizes within individual stores. We therefore believe that the mannequins we sampled in each store were representative of the typical mannequin size, but rating all mannequins in each individual store would have been preferable. A further limitation was that it was only feasible to survey and rate mannequin sizes in national retailers on the high streets of two cities. It may be the case that the size of mannequins in the present study are different to mannequins used in other cities in England, but we presume this is unlikely given that we surveyed national chain retailers. In the present study we found that all female mannequins were slender and it may be the case that if we had sampled specialist shops for customers of larger body size, mannequin size would have been more variable. For example, ‘plus size’ fashion stores exist in some English cities, but no ‘plus size’ stores were sampled in the present study, which is reflective of their small market share in fashion.

### Practical implications & further research

The main implication of the present research is that fashion stores should use more appropriate sized mannequins. Because ultra-thin ideals may encourage the development of body image problems and eating disorders in young people, removing environmental factors that promote ultra-thin ideals is desirable. Future work would benefit from examining the effect that different mannequin sizes have on women’s body image. If being exposed to ultra-thin mannequins has a similarly negative effect on body image as exposure to other forms of ultra-thin media has [28] then this would further support the need for the fashion retail industry to use more appropriate size mannequins. Whether such changes would need to come about through legislation or voluntary action is unclear, but there are a growing number of examples of how public health approaches can target macro level environmental factors in order to prevent body image problems [10]. Given that the prevalence of body image problems and disordered eating in young people is worryingly high, positive action that challenges communication of ultra-thin ideal may be of particular benefit to children, adolescents and young adult females.

## Conclusions

The body size of mannequins used to advertise female fashion is unrealistic and would be considered medically unhealthy in humans.
